# The porcupine *Hystrix parvae* (Kretzoi, 1951) from the Late Miocene (Turolian, MN11) of Kohfidisch in Austria

**DOI:** 10.1007/s12549-024-00616-3

**Published:** 2024-08-03

**Authors:** Gudrun Daxner-Höck, Viola Winkler, Daniela C. Kalthoff

**Affiliations:** 1 Rupertusstraße 16, 5201 Seekirchen, Austria; 2 Department of Geology & Palaeontology, Natural History Museum Vienna, Burgring 7, 1010 Vienna, Austria; 3 Central Research Laboratories, Natural History Museum Vienna, Burgring 7, 1010 Vienna, Austria; 4 Department of Zoology, Swedish Museum of Natural History, Box 50007, 104 05 Stockholm, Sweden

**Keywords:** Hystricidae, Systematics, Morphology, Juvenile individuals, Incisor enamel microstructure, Stratigraphy, Late Miocene, Austria

## Abstract

This paper provides an upgrade of *Hystrix parvae*, which so far is the smallest and oldest extinct species of *Hystrix* s. str. The new data derive from the exceptionally rich new fossil collection of the locality Kohfidisch in Austria, that comprises more than 100 fossils. The detailed descriptions are supported by enamel microstructure analysis of incisors, and x-ray microcomputed images (microCT) of the partial skull, upper and lower jaws and teeth. The tooth pattern and sizes show wide ranges, depending on the ontogenetic stage, tooth position and stage of wear. The deciduous D4/d4 and the permanent lower p4-m3 are identified as low crowned or brachydont. The mean hypsodonty index of m1/2 is 69%. The permanent upper P4-M3 are brachydont to mesodont. The mean hypsodonty index of M1/2 is 114%. The karstic cave and fissure system of Kohfidisch provided an ideal nursery environment as evidenced by numerous deciduous teeth in different ontogenetic stages. The age of the fauna is Late Miocene (lower Turolian, MN11), around 8.6 million years.

**Supplementary Information:**

The online version contains supplementary material available at 10.1007/s12549-024-00616-3.

## Introduction

The fossil record of the genus *Hystrix* ranges from the Late Miocene to the Pleistocene with a spatial distribution from Spain across Europe, the Mediterranean, Turkey, the Caucasus region, Iran, Afghanistan, Siwaliks (India), and as far as China. Today, *Hystrix* species live in temperate and semiarid environments of middle latitudes in Asia, southern Italy and in Northern and Southern Africa.

**Fig. 1:**
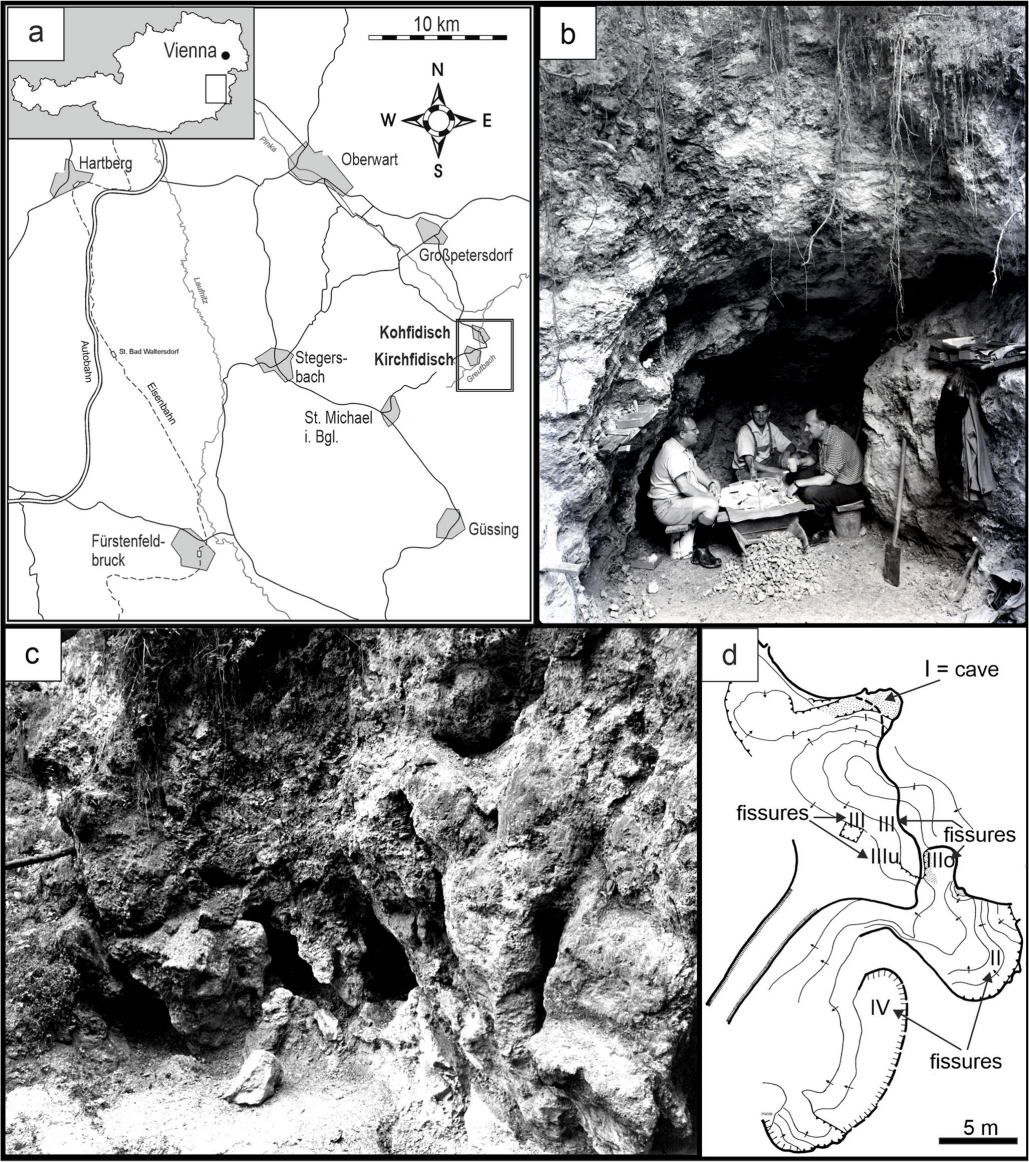
The fossil locality Kohfidisch in Burgenland, Austria**. a** Geographical position of the outcrop Kohfidisch (modified from Bachmayer and Zapfe 1969). **b** Sieving and sorting of washed fossil remains at the entrance of the „Kohfidisch cave“ by F. Bachmayer, H. Zapfe from the Natural History Museum Vienna, and S. Wölfer. **c** Karstic fissures, that were filled by fossiliferous clay. **d** Detailed sketch (Bachmayer and Zapfe 1969) of the outcrop situation showing the main fossil places of the Kohfidisch locality: the cave (Ko-I) and the fissures (Ko-II, Ko-III, Ko-IIIo, Ko-IIIu and Ko-IV). The localization of the outcrop places Ko-Cm, Ko-V and Ko-VI is unknown, it was not marked on the sketch of Bachmayer and Zapfe (1969)

**Fig. 2:**
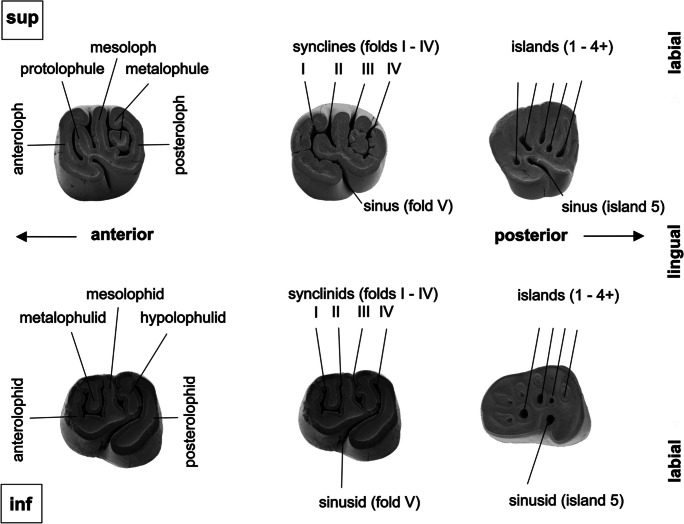
Dental terminology of upper (sup) and lower (inf) cheek teeth of *Hystrix parvae* from Kohfidisch. Modified from Weers (1990) and Daxner-Höck and Höck (2015)

In the course of a revision, Weers and Montoya (1996: 133–134, Pl. 1, figs 1a-1b, 2a-b. 3a-b, 4-5, tab.1) re-described the smaller sized European *Hystrix* findings of the Late Miocene, and attributed them to the species *Hystrix parvae* (Kretzoi, 1951). The species was recognised as the oldest species of *Hystrix* s. str., so far known from four localities, Csákvár (Hungary), Salmendingen (Germany), Crevillente 2 (Spain), and Kohfidisch (Austria) (Weers and Montoya 1996; Sen 1999; Weers and Rook 2003; Sen and Purabrishemi 2010).

Initially, only a few specimens were available from these locations. These are: the holotype of “*Miohystrix” parvae* Kretzoi, 1951 from Csákvár (Hungary), a few teeth described as *Hystrix suevica* Schlosser, 1884 from Salmendingen (Germany), a few teeth from Crevillente 2 (Spain) attributed to *H. suevica* by Montoya (1993), and a small number of specimens from Kohfidisch (Austria) described as *H.* cf. *H. suevica* by Bachmayer and Wilson (1970, 1978, 1980, 1985). Later, a huge *Hystrix*-collection of more than hundred specimens (NHMW 2011/0113/0001-0104) from Kohfidisch, was presented by Daxner-Höck and Höck (2015) and attributed to *H. parvae* (Kretzoi, 1951). The present paper comprises detailed investigations of this new collection from Kohfidisch.

## Locality

The Kohfidisch fossil site (coordinates: E 16°20’39” N 47°08’52”) is located 115 km south-east of Vienna, in the southern part of Burgenland (Fig. 1a). The locality and the first fossils were found in 1955 during geological mapping activities by F. Kümmel at the western slope of the “Hohensteinmaisberg”, south of the villages Kohfidisch and Kirchfidisch (Bachmayer and Zapfe 1969). There, a karstic cave and fissure system was explored in strongly weatherd Devonian limestone and dolomite. The cave and fissures were filled with yellowish-brown fossiliferous clay of the upper Pannonian as evidenced by lithology and fossils. The mollusc fossils are typical of the Pannonian mollusc zone G, and the mammal fossils indicate mammal Zone MN11 of the Late Miocene. These fossil compositions were found from different areas of the cave and fissure system. There is no evidence of any significantly older or younger fossils (see sections „Geological setting“ and „Stratigraphy of Vallesian –Turolian mammal sequences of Austria“).

The excavations were carried out by the Natural History Museum in Vienna in several field campaigns from 1955 to 1984 (Fig. 1b-d). The result of these excavations was an important fossil collection, that is composed of lower vertebrates (Tempfer 2005), turtles, birds, large and small mammals (Bachmayer and Zapfe 1969; Bachmayer and Wilson 1970, 1978, 1980, 1985; Beaumont 1984; Weers and Montoya 1996; Daxner-Höck 2004a, b; Tempfer 2005; Vislobokova 2006; Ziegler 2006; Vislobokova 2007; Daxner-Höck and Höck 2009, 2015).

## Material and methods

### Material

**Fig. 3:**
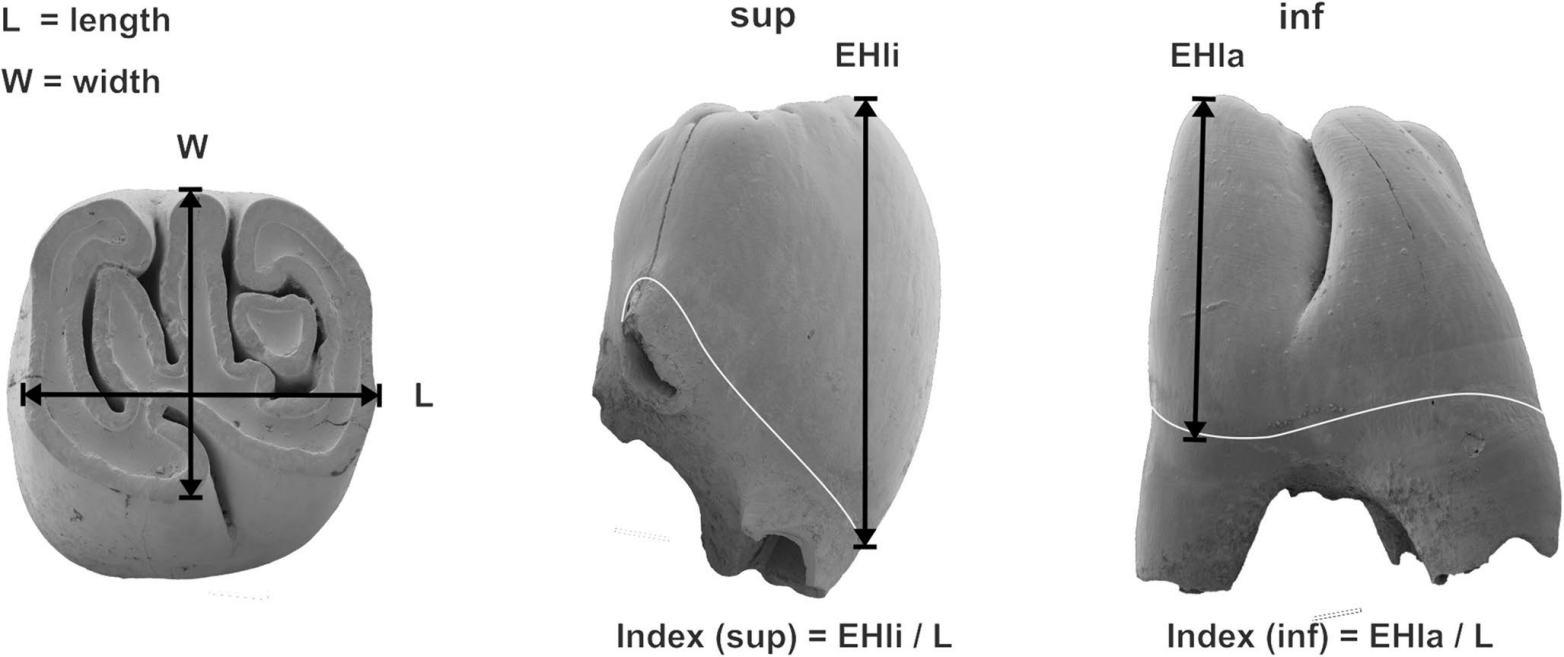
Measurements of cheek teeth. Length (*L*), width (*W*), and enamel height (*EH*). The length (*L*) is measured along the longitudinal axis of the occlusal surface, the value of the width (*W*) is perpendicular to the length

**Fig. 4:**
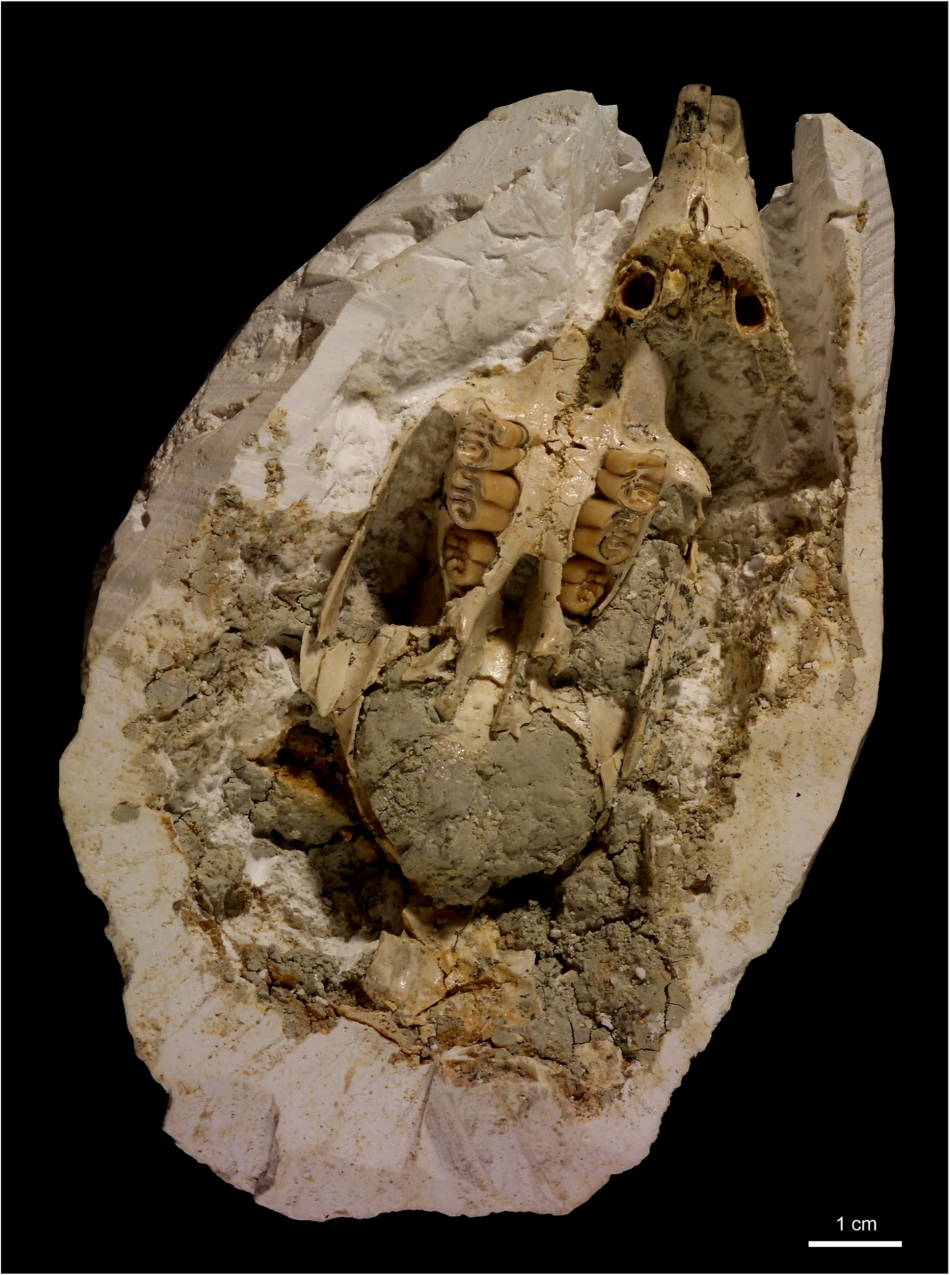
Skull fragment with left and right tooth-rows I2, D4-M2 of a juvenile individual (NHMW 2011/0113/0001) of *H. parvae* from the Late Miocene of Kohfidisch (Austria). Image courtesy of A. Schumacher

**Fig. 5:**
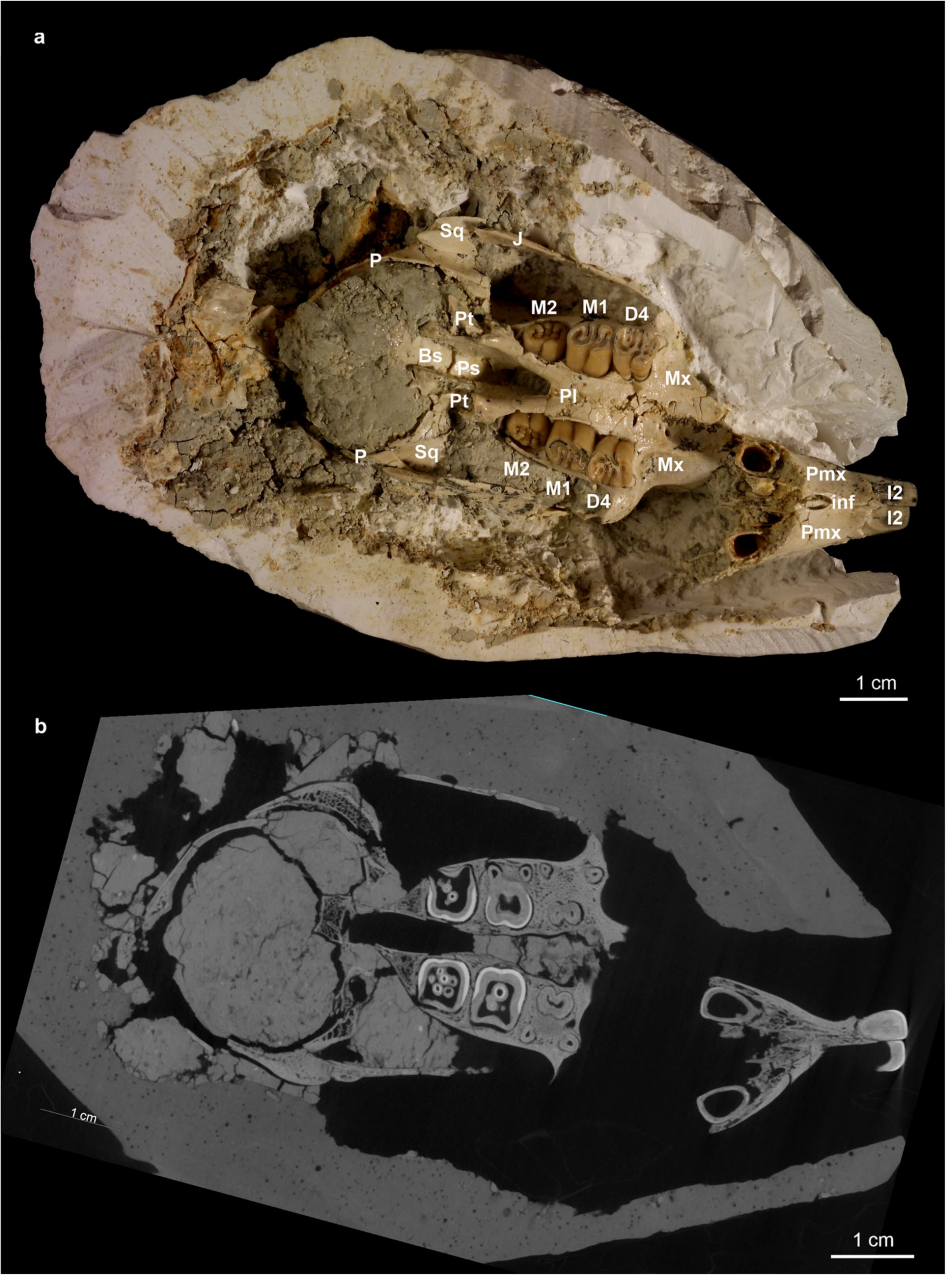
**a** The photo shows the skull fragment with left and right tooth-rows I2, D4-M2 of a juvenile specimen (NHMW 2011/0113/0001) of *Hystrix parvae* (Kretzoi, 1951) from the Late Miocene of Kohfidisch (Austria). **b** The microCT-scan shows the cross section, a few mm towards the dorsal side of the skull. The bones and teeth are comparable with (**a**). The tooth rows show that D4 erupted first, followed by M1 and later M2. Image **a** courtesy of A. Schumacher

**Fig. 6:**
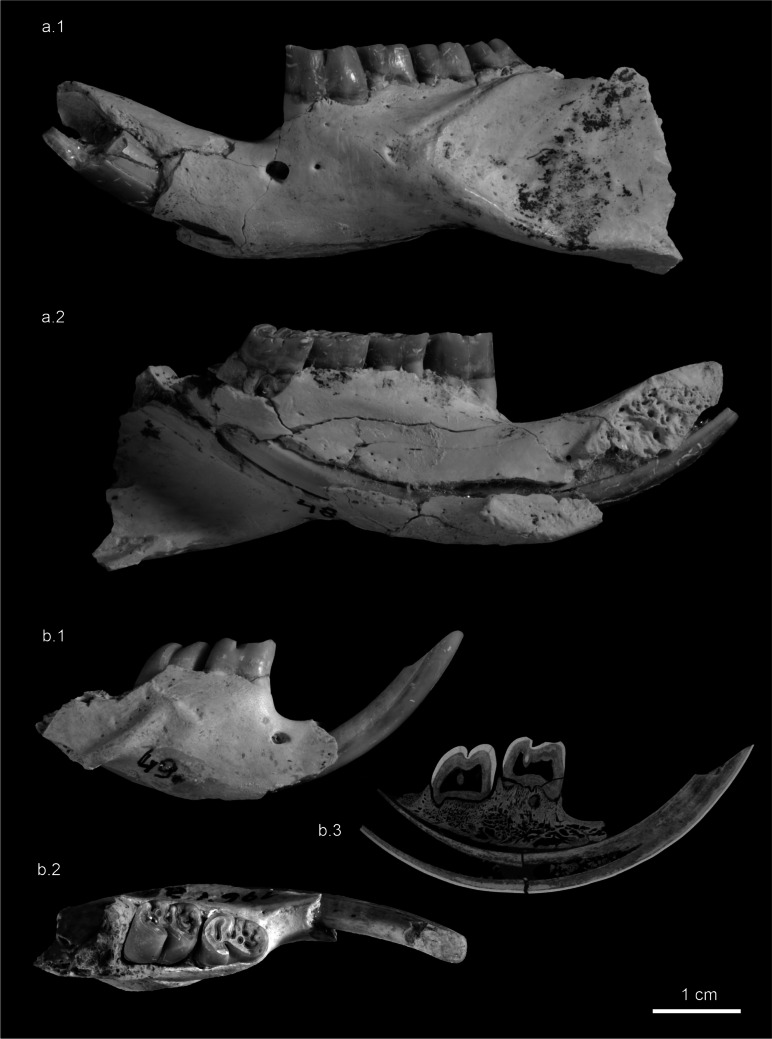
Fragmentary mandibles of *Hystrix parvae* (Kretzoi, 1951) from Kohfidisch (Austria), Late Miocene. **a** left mandible fragment of an adult individual with partial i2, p4-m3 (NHMW 2011/0113/0048), Ko-III. **a.1** labial; **a.2** lingual. **b** right mandible fragment of a juvenile individual with i2, d4-m1 (NHMW 2011/0113/0049), Ko-III. **b.1** labial, **b.2** occlusal, **b.3** microCT-scan of mandible, longitudinal. Images courtesy of A. Schumacher

**Fig. 7:**
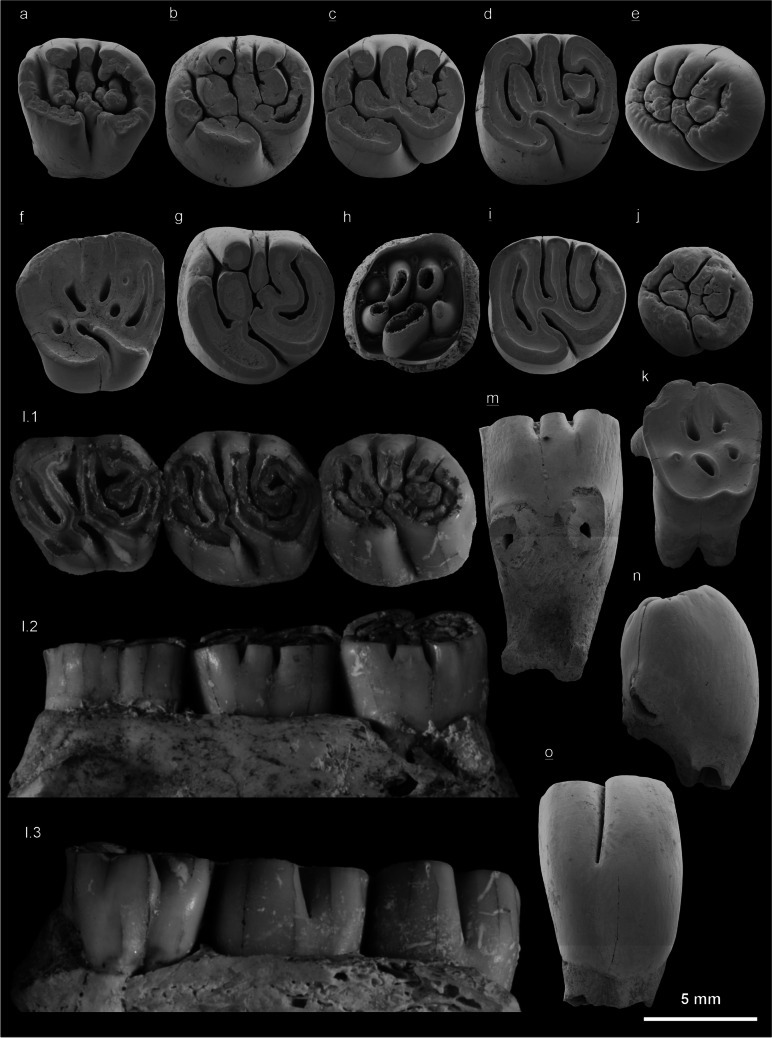
Upper cheek teeth of *Hystrix parvae* (Kretzoi, 1951) from the locality Kohfidisch in Austria (Late Miocene, MN11). **a** left D4 (NHMW 2011/0113/0009), wear-class B, occlusal view, Ko-IIIu. **b** right P4 (NHMW 2011/0113/0018), class B, occlusal view, Ko-I. **c** right M1/2 (NHMW 2011/0113/0020), class B, occlusal view, Ko-I. **d** left M1/2 (NHMW 2011/0113/0025), class C, occlusal view, Ko-Cm. **e** right M3 (NHMW 2011/0113/0044), class A, occlusal view, Ko. **f** right D4 (NHMW 2011/0113/0013), class F, occlusal view, Ko-IIIo. **g** left P4 (NHMW 2011/0113/0014), class C, occlusal view, Ko-IIIu. **h** left M1/2 (NHMW 2011/0113/0029), class A, basal view, Ko-IIIu. **i** right M3 (NHMW 2011/0113/0046), class C, occlusal view, Ko. **j** right M3 (NHMW 2011/0113/0038), class A, occlusal view, Ko-IIIu. **k** left D4 (NHMW 2011/0113/0012), class G, occlusal view, KO-IIIu. **l.1** left D4-M2 (NHMW 2011/0113/0005), class B, occlusal view, Ko. **l.2** left D4-M2 (NHMW 2011/0113/0005), class C, labial view, Ko. **l.3** left D4-M2 (NHMW 2011/0113/0005), class C, lingual view, Ko. **m** right M2 (NHMW 2011/0113/0032), class D, labial view, Ko. **n** left M1/2 (NHMW 2011/0113/0024), class B, mesial view, Ko-IIIo. **o** right M1/2 (NHMW 2011/0113/0035), class B, lingual view, Ko-IIIu. Underlined right (invers), not underlined left. SEM images courtesy of E. Höck, other images courtesy of A. Schumacher

**Fig. 8:**
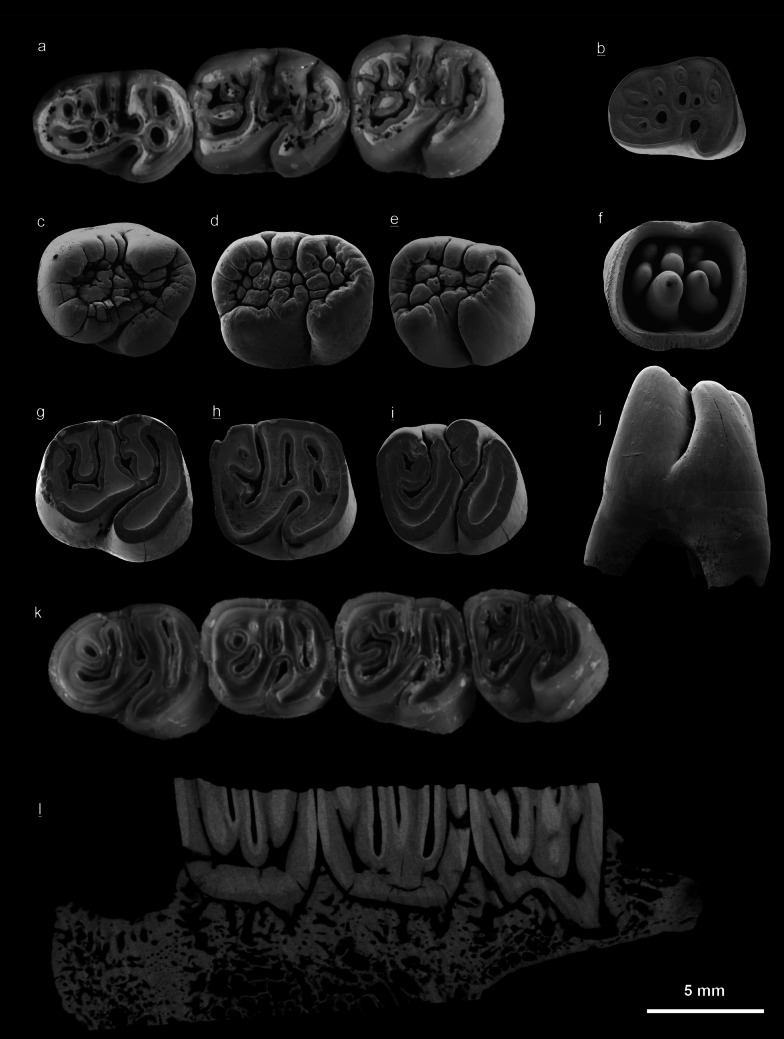
Lower cheek teeth of *Hystrix parvae* (Kretzoi, 1951) from the locality Kohfidisch in Austria (Late Miocene, MN11). **a** left d4-m2 (NHMW 2011/0113/0053), wear-classes S, R, R, occlusal view, Ko-IIIu. **b** right d4 (NHMW 2011/0113/0062), class T, occlusal view, Ko-IIIu. **c** left p4 (NHMW 2011/0113/0066), class O, occlusal view, Ko-IIIo. **d** left m1/2 (NHMW 2011/0113/0078), class O, occlusal view, Ko-I. **e** right m3 (NHMW 2011/0113/0053), class O, occlusal view, Ko-I. **f** left m1/2 (NHMW 2011/0113/0079), class O, basal view, Ko-IIIo. **g** left p4 (NHMW 2011/0113/0071), class P, occlusal view, Ko-I. **h** right m1/2 (NHMW 2011/0113/0070), class O, occlusal view, Ko-I. **i** right m3 (NHMW 2011/0113/0091), class, occlusal view, Ko-IIIu. **j** left p4 (NHMW 2011/0113/0070), class O, labial view, Ko-I. **k** left p4-m3 (NHMW 2011/0113/0048), classes R, S, R, R, occlusal view, Ko-III. **l** right m1-m3 (NHMW 2011/0113/0055), microCT-scan, longitudinal, Ko-I. Underlined right (invers), not underlined left. SEM images courtesy of E. Höck, other images courtesy of A. Schumacher

The collection of the Geological-Palaeontological Department, Natural History Museum in Vienna (NHMW), comprises numerous *Hystrix* fossils from Kohfidisch (Figs. 3, 4, 5, 6, 7 and 8): a partial juvenile skull (with upper tooth-rows), five fragmentary upper jaws and thirteen fragmentary lower jaws with partial tooth-rows of juvenile and adult individuals, almost eighty isolated cheek teeth (8 D4, 5 P4, 32 M1/2, 10 M3, 7 d4, 9 p4, 11 m1/2, 7 m3) and more than seventy fragments of upper and lower incisors. One upper and one lower isolated incisor fragment were used to evaluate the incisor enamel microstructure. The identification of these fragments is unambiguous because of their large size in combination with their enamel microstructure. All fossils and their collection numbers are listed in the material list and in the Appendix. For localisation of the fossils from the karstic fissure and cave system of Kohfidisch we refer on the acronyms Ko, Ko-Cm, Ko-I, Ko-II, Ko-III, Ko-IIIo, Ko-IIIu, Ko-IV, Ko-VI (Fig. 1d and the Appendix).

### Methods

#### Photos and SEM images

Photos of the skull and tooth rows were made at the NHMW. For SEM images of isolated teeth the Philips XL 20 scanning electron microscope of the Biocenter, University of Vienna, was used.

#### MicroCT-analyses

MiocroCT scans were made by using the YXLON FF35 CT system (equipped with a YXLON FXT 225.48 directional beam tube and Y.Panel 4343 CT Csj flat panel detector) of the NHMW. The fossils were scanned with an energy of 90 kV and electric current of 270 µA. The maxilla and mandible fragments and the skull fragment (NHMW 2011/0113/0001) were imaged with energies ranging from 105 kV to 130 kV and electric currents of 140 µA to 270 µA. The skull fragment has a reconstructed isotropic voxel size of 54.4 µm. The isotropic voxel sizes of the teeth range from 10.2 µm to 11.3 µm and of the maxilla and mandible fragments from 11.3 µm to 22.5 µm.

#### Enamel microstructure

Preparations for enamel microstructure analysis were executed following the methodology described in detail in Kalthoff (2000, 2006): small segments of one upper and one lower incisor were embedded in cold moulding epoxy resin (SpeziFix40, Struers). After hardening, transverse and longitudinal sections were prepared by trimming the embedded teeth with a diamond cut-off saw and wet-grinding with Al2O3 grit powder to a minimum grit size of 1000. Samples were subsequently cleaned in an ultrasonic bath, dried with compressed air, and etched for 2–5 seconds with 2 N HCl to make the enamel details visible. Samples were mounted on SEM stubs, sputter coated with gold for two minutes, and studied and documented with a Quanta FEG 650 SEM, located at the Swedish Museum of Natural History, Stockholm, at acceleration voltages of 15 kV and magnifications of x 20 to x 2,500.

#### Dental terminology

For descriptions of *Hystrix*-teeth we follow the combined dental terminology of Daxner-Höck and Höck (2015) and Weers (1990) (Fig. 2). The pentalophodont upper/lower unworn cheek teeth are characterised by lophs/lophids and folds. The folds (synclines, sinus, synclinids, sinusid) between the lophs/lophids are open at the lingual/labial tooth-face, respectively. With increased wear they disappear and become islands (enclosed by enamel).

#### Measurements

The measurements of length, width, and height were taken as shown in Fig. 3. The length (L) and width (W) are the maximum values of the cheek teeth measured along the occlusal surface, and depend on the actual stage of wear. The tooth height is also depending on wear, it is measured as the enamel height (EH) of the crown. The values of upper teeth are measured lingually (EHli), those of lower teeth labially (EHla). The enamel height (EH) is measured from the occlusal surface to the crown/root boundary (“linea sinuosa”), termed by Rabeder (1981). The hypsodonty index (I) shows the relation of the enamel height and the length (index = EH/L) and is described in %. Index sup = EHli/L, index inf = EHla/L. For measurements of the EH only isolated teeth were used, because the enamel/dentine-border is not exactly visible on in situ tooth-rows.

#### Wear classes

For interpretation of the occlusal pattern of cheek teeth from *Hystrix* we use the wear classes, defined by Weers (1990) on the basis of the modern *Hystrix brachyura*. This classification method was modified for fossil species by Weers and Rook (2003), and again slightly adapted for *H. parvae* from Kohfidisch in the present work. Permanent cheek teeth from the upper dentition show up to four labial folds (synclines 1– 4) and one lingual fold (sinus), those of the lower dentition show also up to four labial folds (synclinids 1–4) and one lingual fold (sinusid). With increased wear the folds successively close towards the tooth margin, and become islands. Depending on the number of folds and islands, seven wear-classes (A – G) of upper cheek teeth, and six wear classes (O – T) of lower cheek teeth are distinguished from *H. parvae*, as shown in Table 1.

**Table 1:** Seven wear-classes of upper and six of lower cheek teeth of *Hystrix parvae* from Kohfidisch are distinguished*.* The upper cheek teeth show wear-classes A (unworn) to G (worn down), and lower cheek teeth classes O (unworn) to T (worn down)

wear class	number of folds	number of islands
upper cheek teeth (D4, P4, M1-3)		
A	5	0
B	4–5	0
C	3–4	1
D	2–4	2
E	2–3	3
F	1	3–5
G	0	4–5
lower cheek teeth (d4, p4, m1-3)		
O	2–5	0-2
P	2–3	0
Q	2–3	1
R	2-3	2–3
S	1-3	2–6
T	0	0–6

In order to facilitate comparisons right side teeth (except Fig. 6b.1.-3.) are figured as mirror images (as if they were from the left side), and their figure letters are underlined (e.g. Fig. 7b shows the inversed P4 from the right body side).

### Abbreviations

NHMW: Natural History Museum Vienna
MN: Mammal Neogene Zones (Mein 1975)
sup: upper cheek teeth (D4, P4, M1, M2, M3)
inf: lower cheek teeth (d4, p4, m1, m2, m3)
I2: upper incisor
i2: lower incisor
n: number of specimens
L: length
W: width
EH: enamel height
EHli: lingual enamel height of upper cheek teeth
EHla: labial enamel height of lower cheek teeth

## Systematic palaeontology

Family Hystricidae Fischer von Waldheim, 1817

Genus *Hystrix* Linnaeus, 1758

**Fig. 9:**
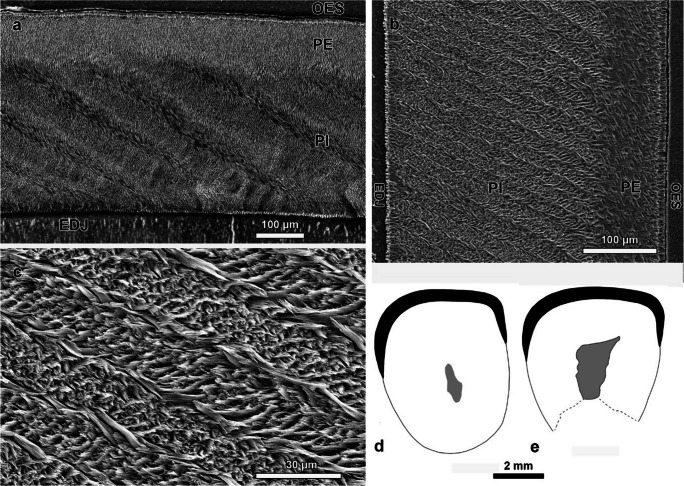
Incisor enamel microstructure and incisor cross sections of *Hystrix parvae* (Kretzoi, 1951) from the locality Kohfidisch in Austria (Late Miocene, MN11)*.* **a**–**c** scanning electron micrographs of lower and upper incisor; **a** transverse section of the lower incisor (NHMW 2011/0113/0102); **b** longitudinal section of the upper incisor (NHMW 2011/0113/0101); **c** detail of b showing the multiserial Hunter-Schreger bands of the portio interna; **d** cross section of lower incisor; **e** cross section of upper incisor. Both cross sections are shown as left side and drawn to scale. Abbreviations: *EDJ* enamel–dentine junction; *OES* outer enamel surface; *PE* portio externa; *PI* portio interna

*Hystrix parvae* (Kretzoi, 1951)

(Figures 4, 5, 6, 7, 8, 9, Tables 1, 2, 3)

**Table 2:** Measurements of *Hystrix parvae* from Kohfidisch. Length (*L*) and width (*W*) of the occlusal surface of upper (*sup*) and lower (*inf*) cheek teeth, and the lingual and labial enamel height of upper (*EHli*) and lower (*EHla*) cheek teeth. Only measurements taken from isolated teeth were included in this table

object	L (length occlusal)			W (width occlusal)			EHli (enamel hight lingual)		
sup.	range	mean	n	range	mean	n	range	mean	n
D4	6.00 – 7.50	7.10	13	4.20 – 6.20	5.25	12	2.50 – 6.50	4.65	6
P4	7.30 – 7.70	7.50	4	5.50 – 7.00	6.50	4	7.00 – 8.00	7.50	4
M1/2	5.80 – 8.00	7.20	32	4.50 – 8.00	5.30	32	7.00 – 9.00	8.50	14
M3	5.50 – 7.10	6.50	11	4.00 – 7.00	5.45	10	4.00 – 8.00	6.20	9
	L (length occlusal)			W (width occlusal)			EHla (enamel hight labial)		
inf.	range	mean	n	range	mean	n	range	mean	n
d4	6.10 –7.90	7.20	9	4.30 – 5.70	5.30	9	2.00 – 4.00	2.40	5
p4	6.50 – 8.30	7.50	10	4.50 – 6.70	6.00	10	3.00 – 6.70	5.50	8
m1/2	6.50 – 8.00	7.20	28	4.50 – 7.00	5.20	27	3.00 – 6.00	5.00	11
m3	5.80 – 6.90	6.30	12	4.00 – 6.50	4.75	12	4.00 – 8.00	4.50	6

**Table 3:** Distribution of 57 upper and 53 lower cheek teeth of *Hystrix parvae* from Kohfidisch to wear classes A–G for upper, and O–T for lower cheek teeth

upper cheek teeth	A	B	C	D	E	F	G	n = 57
D4	1	2		3	1	3	3	n = 13
P4		2	2					n = 4
M1			2	3	1			n = 6
M1/2	4	6	6	2		1	2	n = 21
M2	2		1		1	1		n = 5
M3	3	2	2				1	n = 8
lower cheek teeth	O	P	Q	R	S	T		n = 53
d4		1		2	2	3		n = 8
p4	4	3	1	2	2			n = 12
m1	1			2	1			n = 4
m1/2	5	1	1	2		1		n = 10
m2	4			4				n = 8
m3	8	1	1	1				n = 11

1951 *Miohystrix parvae* n. gen. n. sp. Kretzoi: 407.

1970 *Hystrix* cf. *H. suevica* Bachmayer and Wilson: 579–580.

1978 *Hystrix* cf. *H. suevica* Bachmayer and Wilson: 157.

1980 *Hystrix* cf. *H. suevica* Bachmayer and Wilson: 383.

1993 *Hystrix suevica* Montoya: Pl. I-4, I-5.

1996 *Hystrix* cf. *suevica* Daxner-Höck: 4.

1996 *Hystrix parvae* Weers and Montoya: 134–141, pl.1, figs. 2a, 2b, 3a, 3b, tabs. I–III.

1999 *Hystrix parvae* Sen: 428–433, tabs. 42.1–42.2, figs. 42.2–42.4.

2010 *Hystrix parvae* Sen and Purabrishemi: 239, 243–247, fig. 4.

2015 *Hystrix parvae* Daxner-Höck and Höck: 62–64, fig. 23, Pl. 69.1, Pl. 70.1–4, Pl. 71.1–16, Pl. 72.1–12, Pl. 73.1–4.

**Holotype:** Fragment of the right mandibular ramus with p4-m2 and a small part of the i2 (F.I.V.6006) of *Miohystrix parvae* Kretzoi, 1951 from Csákvár.

**Paratypes:** Fragments of an upper and a lower incisor (F.I.V. 6007) of *Miohystrix parvae* Kretzoi, 1951 from Csákvár.

**Type locality:** Csákvár, Esterházy–Cave (Baracházar), County Fejér, Hungary, Late Miocene, early Turolian (MN11).

**Other occurrences**: Salmendingen (Germany), Crevillente 2 (Spain) and Kohfdisch (Austria).

**Stratigraphical and geographical range:** Late Miocene, Turolian, MN11, Europe

**Material:** A skull fragment with left and right tooth-rows I2, D4-M2 of a very young individual (NHMW 2011/0113/0001), 5 maxilla fragments with partial tooth-rows of juvenile and adult individuals (NHMW 2011/0113/0002–0005, 2015/0098), 8 D4 (NHMW 2011/0113/0007–0013, NHMW 2011/0113/0097), 4 P4 (NHMW 2011/0113/0014–0015, NHMW 2011/0113/0017–0018), 23 M1/2 (NHMW 2011/0113/0006; NHMW 2011/0113/0019–0037, NHMW 2011/0113/0094–0096), 10 M3 (NHMW 2011/0113/0038–0047), 13 fragmentary mandibles with partial tooth-rows (NHMW 2011/0113/0048–0057, NHMW 2011/0113/0074, NHMW 2011/0113/0085, NHMW 2011/0113/0099), 7 d4 (NHMW 2011/0113/0058–0063, NHMW 2011/0113/0068), 10 p4 (NHMW 2011/0113/0064–0067, NHMW 2011/0113/0069–0073, NHMW 2011/0113/0016), 11 m1/2 (NHMW 2011/0113/0074–0084, NHMW 2011/0113/0086), 7 m3 (NHMW 2011/0113/0087–0093) and incisor fragments (NHMW 2011/0113/0100–0104). For localisation of the specimens in the outcrop see Fig. 1d. and Appendix.

**Description of the fragmentary skull:** The fragmentary juvenile skull (NHMW 2011/0113/0001) is still partly imbedded in plaster, and displays both tooth-rows (I2, D4, M1 and M2 left and right) in ventral view (Figs. 4, 5). The nasals and the dorsal, lateral and posterior parts of the skull are not preserved. The premaxilla bones (Pmx) are isolated from the maxilla (Mx) along the maxilla-premaxilla suture, and the premaxilla bones and upper incisors (I2) are shifted towards the left side. The preserved parts of the skull are: left and right premaxilla (Pmx), maxilla (Mx), and parts of the pterygoid (Pt), squamosum (Sq), jugal (J), the palatine (Pl), presphenoid (Ps), basisphenoid (Bs) and smaller parts of the parietale (P). Due to the early ontogenetic stage all bones are very thin and fragile. The left and right tooth rows, the I2, D4, M1 and M2, are already erupted. The milk premolar (D4) and the first permanent molar (M1) are rooted and already in function, however, the second M2 is not yet fully erupted. The embryonal P4 and M3 are not yet developed/erupted, they are not visible in the microCT- scan.

All available teeth are in early wear stage (wear-classes A-B) indicating a very young ontogenetic phase. The zygomatic arch from the right body side, formed by the zygomatic process of the maxilla (Max), the jugal (J), and the zygomatic process of the squamosal (Sq) is preserved, the left one is in bad condition. The diastema between I2 and D4 is rather short. The incisive foramen (inf) is small, oval shaped and located immediately anterior to the maxilla-premaxilla suture. The left and right tooth rows converge towards the posterior. The transverse maxillo-palatine suture is aligned with the middle part of M1. The posterior border of the hard palate is aligned with the M2. The fragmentary skull suggests a total length of about 8 cm.

**Description of the mandible**: The collection comprises thirteen mandible fragments of juvenile and adult individuals, partly with complete, others with incomplete tooth rows. Both, the angular and coronoid processes are broken. The symphysis of the horizontal ramus extends posteriorly to below the anterior root of p4 (Fig. 6a.2). The diastema is as long as 3–4 teeth of adult individuals (Fig. 6a) but shorter in juvenile mandibles (Fig. 6b). The diastema is asymmetric, concave, deepening posteriorly. The mental foramen is located below the anterior root of d4/p4. The masseteric fossa extends anteriorly to below the m1. The juvenile mandible is built up by very thin bone (Fig. 6b), and is distinctly smaller than the adult specimen (Fig. 6a).

**Characterisation of cheek teeth from Kohfidisch**: The teeth are pentalophodont and can be described as brachydont to mesodont. The occlusal surface is flat. The occlusal pattern and tooth sizes show wide ranges, depending on the ontogenetic stage, tooth position and wear stage.

The occlusal surface of newly erupted, almost or completely unworn teeth are wrinkled, and sometimes no roots are yet developed. This pattern is typical of wear-classes A-B of upper cheek teeth (Fig. 7a, e, j), and wear-classes O-P of lower cheek teeth (Fig. 8c, d, e). With wear the wrinkles disappear and dentine becomes visible on the occlusal surface. Moderately worn upper cheek teeth are classified to wear-classes C-F**,** and the more worn lower cheek teeth to wear-classes Q-S. The final wear-classes G of upper cheek teeth (Fig. 7k) and T of lower cheek teeth (Fig. 8b) are characterised by enamel islands only, and by very low crown heights.

The length/width measurements of P4/p4 are largest, that of M3/m3 smallest (Table 2). The The D4/d4 are rather small and low crowned, some have not yet developed roots. They show the lowest mean hypsodonty index. In contrast, permanent cheek teeth (P4-M3) of young adult individuals – specifically the unworn or slightly worn teeth – show the highest mean hypsodonty index values of *H. parvae* from Kohfidisch. One reason for the high mean index values is the large number of almost unworn upper cheek teeth of the *Hystrix* collection from Kohfidisch. The second, and main reason is the elongated and strongly curved lingual wall of upper permanent cheek teeth, described as “partial hypsodonty” (Tobien 1963; Koenigswald 2011). The hypsodonty index of lower molars (m1-m3) is regularly lower than that of upper antagonists (Table 4).

**Table 4:** The hypsodonty index expresses the enamel height as a percentage of the length of the occlusal surface. The index of upper teeth (index sup) shows the relationship of the lingual enamel height and the length of the occlusal surface (EHli/L). The index of lower cheek teeth (index inf) shows the relationship of the labial enamel height (EHla/L) and the length of the occlusal surface. n=number of specimens

upper cheek teeth	index sup = EHli/L		n
	range	mean	
D4	38 – 81 %	70 %	6
P4	93 – 105 %	96 %	4
M1/2	97 – 145 %	118 %	19
M3	61 – 166 %	102 %	9
lower cheek teeth	index inf = EHla/L		n
	range	mean	
d4	27 – 57 %	33 %	5
p4	40 – 95 %	73 %	7
m1/2	40 – 83 %	69 %	11
m3	62 – 137 %	75%	6

### Description of the cheek teeth

**D4** (Fig. 7a, f, k, l.1; Tables 2, 3, 4) is of trapezoidal outline, the labial side is longer than the lingual side. D4 is low crowned compared with P4. The forwards-directed sinus persists to a late stage of wear (class F). The sinus of one unworn specimen is continuous with the first fold, it is continuous with the second fold in another specimen. Four lophs, the anteroloph, protolophule, mesolph and posteroloph are developed. The metalophule (5th loph) is of variable shape, it is short or curved backwards and fuses with the posteroloph. Consequently, the folds are of variable shape, too. Three D4 of the Kohfidisch collection are unworn or almost so, showing four folds and no enamel island (classes A–B), four specimens in moderate wear stages show 3–4 folds and 2–3 islands (classes D–E), six specimens are almost completely worn down showing 0–1 fold and 4–6 islands (classes F–G). The length of D4 ranges from 6.00 to 7.50 mm (mean 7.10 mm). The D4 is lowest of all upper teeth, with a hypsodonty index ranging from 38 to 81 % (mean 70 %). The D4 has three to four roots: two labial roots, and one wide lingual root with two tips, or two lingual roots close to each other. Completely unworn teeth do not yet have developed roots.

**P4** (Fig. 7b, g; Tables. 2, 3, 4) is the largest among upper cheek teeth, it is widest in the protocone-anteroloph-paracone part. The sinus of one unworn specimen is continuous with the first fold, it is continuous with the second fold in another specimen. P4 is robust and much higher than D4. The occlusal surface shows a maximum of length and width measurements in a medium wear stage of P4. Two newly erupted P4 have wrinkled enamel-surfaces and five folds but no island (class B), and two slightly worn specimens show four folds and one island (class C). The crown is higher on the lingual face than on the labial one. The lingual face is convex. The P4 is longer than wide, its length ranges from 7.30 to 7.70 mm (mean 7.50 mm), the width ranges from 5.50 to 7.00 mm (mean 6.50 mm). The hypsodonty index ranges from 93 to 105% (mean 96 %), depending on the stage of wear. The P4 have three to four roots of irregular position.

**M1/2** (Fig. 7c, d, h, l.1-3, m, n, o; Tables 2, 3, 4) is almost as long as P4 in occlusal pattern, but not as wide as P4. Because of the variable dental pattern isolated M1 and M2 cannot be separated from each other with confidence. The sinus of a few specimens is continuous with the first fold, in other specimens with the second fold, however, in most cases the sinus is not connected with any labial fold nor loph. The large number of M1/2 (32 specimens) provides a wide range of wear-stages (A–G), and strongly differing measurements. There are four unworn teeth with completely wrinkled occlusal surfaces (class A), six teeth are slightly worn showing five folds (class B), nine teeth in a low wear -stage show four folds and only one island (class C), seven more strongly worn specimens show 2–4 folds and 2–3 islands (classes D–E), and four strongly worn teeth show 0–1 fold and 3–6 islands (classes F–G). Very young teeth without developed roots (Figs. 7h, 8f) allow a view into the crown from below. There, the folds/islands extend to the base of the crown, reminiscent of fingers of a glove. The length of the occlusal surface ranges from 5.80 to 8.00 mm (mean 7.20 mm), the width from 4.50 to 8.00 mm (mean 5.30 mm) and the height 7.00 to 9.00 mm (mean 8.50 mm). Because of the convex lingual face, the lingual enamel height exceeds the labial one. The hypsodonty index of nineteen specimens ranges from 97 to 145 % (mean 118 %). M1/2 have three roots, two labial roots and one wide lingual root split in two tips.

**M3** (Fig. 7e, i, j; Tables 2, 3, 4) is similar to M1/2 in molar pattern, but narrows in its posterior part, and generally differs by smaller sizes. One extremely small isolated tooth of wear-class A is a juvenile tooth without developed roots (Fig. 7j). Additionally, there are two unworn M3 with a wrinkled crown surface (class A), two slightly worn teeth have 4-5 folds (class B), two M3 have four folds and one island (class C), only one M3 is strongly worn down, showing six islands but no folds (class G). The length of the occlusal surface ranges from 5.50 to 7.10 mm (mean 6.50 mm), the width ranges from 4.00 to 7.00 mm (mean 5.45 mm). The hypsodonty index of nine isolated M3 ranges from 61 to 166 % (mean 102 %). M3 has three roots.

**d4** (Fig. 8a, b; Tables 2, 3, 4) is widest in its posterior and narrow in the anterior part. The sinusid is directed backwards and persists from wear-class O to S. There is only one slightly worn specimen showing two lingual folds reaching the margin of the tooth, but no island is present (class P), four specimens with prograding wear show 2–3 folds and 2–6 islands (classes R–S), and three specimens are almost worn down showing no fold but 6-8 islands (class T). The length of d4 ranges from 6.10 to 7.90 mm (mean 7.20 mm). The d4 is lowest of all lower cheek teeth with a hypsodonty index of five 27–57% (mean 33 %). The d4 has three roots, one anterior and two posterior roots.

**p4** (Fig. 8c, g, k, j; Tables 2, 3, 4) is the largest of the lower teeth, it is wider than m1/2. It is widest in the hypoconid-entoconid region. In some unworn or slightly worn specimens the sinusid is continuous with the posterior fold. Eight moderately worn teeth show 3-4 folds and 0–1 islands (classes O, P, Q), four strongly worn teeth show 1–2 folds and 3–5 islands (classes R–S). The length of p4 ranges from 6.50 to 8.30 mm (mean 7.50 mm). The hypsodonty index of seven measured teeth is 40–95% (mean 73%). The p4 have three to four roots, one anterior with split tips and two posterior roots.

**m1/2** (Fig. 8a, d, f, h, k; Tables 2, 3, 4) is rectangular in occlusal outline, and has rounded corners. The occlusal pattern is similar to p4 but wider in the anterior part. In early wear stages the folds 1-3 are lingually open, but close with increasing wear. Some unworn/slightly worn specimens show the connection of sinusid and fold 3. Twelve unworn or moderately worn m1–m2 show 3–4 folds and 0–1 islands (classes O, P, Q), nine strongly worn specimens show 1–3 folds and 3-5 islands (classes R–S). Only one m1/2 is completely worn down, as evidenced by the low crown and 6 islands but no fold (class T). The length ranges from 6.50 to 8.00 mm (mean 7.20 mm). The hypsodonty index of eleven measured teeth is 40– 83% (mean 69%). The m1/2 has two anterior and two posterior roots.

**m3** (Fig. 8e, i, k; Tables 2, 3, 4): The occlusal pattern is similar with m1/2, but the tooth is smaller, and narrows posteriorly. The folds 3 and 4 are frequently continuous. Nine m3 are unworn or almost so, showing 3–4 folds but no islands (wear-classes O–P), the remaining two moderately worn specimens have three folds and 1-2 islands (classes Q–R). The length of m3 ranges from 5.80 to 6.90 mm (mean 6.30 mm). The hypsodonty index of six measured m3 ranges from 62 to 137% (mean 75%). The m3 has three roots, two anterior ones and one in posterior position.

**Enamel microstructure of incisors**: The overall incisor shape is roundish-oval and the outer enamel surface is smooth (Fig. 9d-e). In the lower incisor, the enamel is flattened in the central part of the band and extends more to the lateral than to the lingual side (Fig. 9d). In the upper incisor, the enamel is slightly convex and extends equally to the lateral and lingual sides (Fig. 9e). A lateral longitudinal fold is absent in both incisors. The lower incisor cross sectional shape (incisor width divided by length; Rybcynski 2007: appendix B, character 54) is 0.79 and therefore attributed to character state 1.

The schmelzmuster of the lower and upper incisors of *Hystrix parvae* is very similar.

The enamel is thick and measures about 433 µm both in the lower and the upper incisor (average of 10 measurements each). The schmelzmuster is two-layered with a thicker inner portion (portio interna, PI; ca 75% of total enamel thickness) with multiserial Hunter-Schreger bands (HSB) and a thinner outer portion (portio externa, PE; ca 25% of total enamel thickness) with radial enamel (Fig. 9a-c). The junction between the two portions is not sharp and somewhat diffuse. With about 28° for the lower incisor and 24° for the upper, the inclination of the HSBs is low. Individual HSB consist of 4-6 prisms and narrow transition zones are common (Fig. 9c). The overall HSB arrangement appears to be more regular in the lower incisor with less converging or diverging bands compared to the upper one. The interprismatic matrix (IPM) is of medium thickness and well discernable. It is arranged mostly parallel but also at acute angle to the prism long axes. In the radial enamel of the PE, prisms are steeply inclined and at large angle or rectangular to the IPM. The lower incisor has a thin layer of prismless enamel (PLEX). Individual prisms are laterally flattened in the HSB and lanceolate shaped in the radial enamel.

## Discussion

The entire collection of *Hystrix* from Kohfidisch comprises fossil remains of different ontogenetic stages, deciduous (D4 and d4) and permanent cheek-teeth. They are either unworn (with wrinkled enamel surface and partly without developed roots), or worn teeth of different wear-classes. Some are completely worn down (very old permanent or strongly worn deciduous teeth). Most striking is the high frequency of juvenile individuals, about 50 %. There, a minimum of 10 juvenile and 10 adult individuals is evidenced by 10 left D4 and 10 left p4.

The highest length and width values were measured from teeth at a low wear stage (mainly P4, p4 and M1-3), the smallest values are from completely worn teeth. The height of upper teeth was measured as lingual enamel height (EHli), which is constantly higher than the labial enamel height (EHla) measured from lower teeth (Table 2).

The term “hypsodonty” describes the increasing tooth height during the ontogenetic or phylogenetic development. Height increase of teeth starts from brachydont (low crowned), and can lead to hypsodont (high crowned) or euhypsodont (rootless) teeth. Many terms, used by different authors, characterise the increasing height between brachydont and hypsodont, or between different types of hypsodont teeth. The hypsodonty index indicates the relation of height/length or height/width. However, the usage of all these terms is variable and can sometimes cause misunderstandings.

The NOW (New and Old Worlds) fossil mammal database uses the hypsodonty index, calculated from the relation between height and length of the second upper or lower molars (Koenigswald 2011, p. 68). According to that, brachydont teeth have an index < 0.80 (< 80%), mesodont teeth an index between 0.80 and 1.20 (80 to 120%), and hypsodont teeth an index > 1.20 (> 120%).

We also use the height/length relationship and calculate the index values (max, mean and min) for all tooth positions (Table 4). According to that, almost all milk teeth (D4/d4) and the main part of lower permanent teeth (p4-m3) of *H. parvae* from Kohfidisch are brachydont, whereas most of upper permanent teeth (P4-M3) are mesodont. Only very few isolated unworn M1/2, M3 and m3 exceed the value of 120%, and therefore are considered hypsodont.

*H. parvae* was identified as the oldest and smallest species of the genus *Hystrix* s. str. based on a small number of fossils (Weers and Montoya 1996; Sen 1999; Weers and Rook 2003; Sen and Purabrishemi 2010). The present data from Kohfidisch (Tables 2, 3, 4 and Figs. 4, 5, 6, 7, 8) allow for a re-evaluation of *H. parvae.* The available data of *H. parvae* from Csákvár (Hungary), Salmendingen (Germany) and Crevillente 2 (Spain) are within the ranges of the Kohfidisch specimens.

For comparisons with other *Hystrix* species the mean values of length, width and height (Table 2) of the specimens from Kohfidisch, and the hypsodonty index (Table 4) are considered:

- mean length of M1/2 (7.20 mm) and m1/2 (7.20 mm),
- mean width of M1/2 (5.30 mm) and m1/2 (5.20 mm),
- mean enamel height (li) of M1/2 (8.50 mm) and mean enamel height (la) of m1/2 (5.00 mm),
- mean hypsodonty index of M1/2 (1.18 = 118%) and m1/2 (0.69 = 69%)

It was already demonstrated in earlier publications (Weers and Montoya 1996; Sen 1999; Weers and Rook 2003; Sen and Purabrishemi 2010), that stratigraphically younger European *Hystrix* species show higher measurements, but do not significantly change dental morphology. Consequently, size and the hypsodonty index provide more reliable tools for species differentiation than dental morphology. The cheek teeth of *H. primigenia* (Turolian, MN11–13) are larger than those of *H. parvae*, however, the mean EH/L index is < 100 to 150% (mesodont–hypsodont). A moderate height increase is evident from middle Turolian to early Ruscinian (MN12–15), and the EH/L index of *H. depereti* is > 150% (hypsodont). However, a sudden and significant increase of hypsodonty occurred during the Late Pliocene and the Pleistocene as indicated by *H. refossa* (EH/L index is 150 to 250%; hypsodont) (Weers and Montoya 1996; Sen 1999; Weers and Rook 2003; Sen and Purabrishemi 2010; Fejfar and Sabol 2022).

We also recognised that *Hystrix* species described from the Caucasus region and from various localities in Asia (Afghanistan, Iran, India and China) are larger in size and stratigraphically younger than *H. parvae.* They follow in most cases the trend of increasing tooth sizes with time (Late Miocene, MN11 to Late Pliocene, MN14), with one exception, *H. kayae* (Turkey).

The two older Asian species with mesodont to hypsodont, medium sized teeth are *H. lufengensis* from Lufeng (China; Late Miocene, early to middle Turolian, MN11-12; Wang and Qiu, 2005) and *H. sivalensis* from the Sivaliks (India; Late Miocene, middle Turolian, MN 12; synonym with *H. primigenia* according to Weers and Rook 2003).

*H. aryanensis* from Molayan (Afganistan) and Ivand I (Iran); Late Miocene, early to middle Turolian, MN 11-12; Sen and Purabrishemi 2010), *H. gansuensis* from Linxia and Lufeng (China; Late Miocene to Early Pliocene; Wang and Qiu 2002; Flynn and Wu 2017), and *H. previrostra* from the Linxia Basin (China; Late Miocene to Early Pliocene; Wang and Qiu 2020) have medium sized, mesodont to hypsodont cheek teeth. The tooth height of *H. previrostra* is lowest of the three species.

*H. caucasica* from Kosyakino (Russia*,* Early Pliocene, MN14; Lopatin et al. 2003) is based only on one P4, which is described as larger than P4 of other Miocene-Pliocene species, but rather low crowned.

*H. kayae* from Corakyerler (Turkey; Late Miocene, late Vallesian, MN10 or earliest Turolian, MN 11; Halaclar et al. 2023) has medium sized, brachydont to mesodont cheek teeth (mean hypsodonty index < 100 %). The length/width measurements significantly exceed the measurements of *H. parvae* of the lower Turolian (MN11)*,* however, they agree with the overlapping size range of *H. primigenia* and *H. depereti,* and fit with values of Turolian taxa from Asia*.* These measurements and the mean hypsodonty index (< 100%) indicate metric affinities with *H. primigenia* (Europe) and *H. lufengensis* (China). The assumed late Vallesian or early Turolian age of *H. kayae* (Halaclar et al. 2023) raise doubts on the stratigraphy of the Turkish locality.

### Enamel microstructure

The enamel microstructure in different *Hystrix* species have been described before (Martin 1992; Koenigswald and Mörs 2001; Mörs and Hugueney 2017). *Hystrix parvae*, however, as the stratigraphically oldest representative of the genus in Europe has not yet been studied in detail. The species is considered an immigrant from Asia (Sen and Purabrishemi 2010) and we can show here that it arrived in Europe with typical, multiserial Hunter-Schreger bands (Fig. 9). In fact, the enamel and schmelzmuster characters of *H. parvae* are very similar to those in the Late Miocene *H. sivalensis* described in Martin (1992), as well as to fossil and extant *Hystrix* and extant *Atherurus* (Martin 1992; Koenigswald and Mörs 2001; Mörs and Hugueney 2017). Only the HSBs curvy course in lower incisors of extant *H.* cf. *cristata* deviates from the “usual” *Hystrix* features (Koenigswald and Mörs 2001: fig. 5a). Within Hystricognathi, the incisor schmelzmuster in Hystricidae is regarded as rather primitive because the IPM is prism-parallel or at acute angle to the prisms (Martin 1992). We consider schmelzmuster to be conservative within *Hystrix* with no evolutionary changes since the Miocene.

## Geological setting

Development and stratigraphy of the Late Miocene mammal faunas of Austria are closely related with the history of the Northern Alpine Foreland Basin, the Vienna Basin and the Pannonian Basin, and with the origin and development of Lake Pannon (Harzhauser et al. 2004; Daxner-Höck et al. 2016)

At the Middle/Late Miocene-transition (= Sarmatian/Pannonian boundary), i. e. at about 11.6 Ma, a glacioeustatic sea-level drop caused the final disintegration of the Paratethys Sea (Harzhauser et al. 2004). The Paratethys split geographically into the Eastern Paratethys and, west of the Pannonian basin system, Lake Pannon arose (Magyar et al. 1999; Harzhauser et al. 2004; Harzhauser and Mandic 2008). In Austria, Lake Pannon covered the northern and southern Vienna Basin and the Austrian part of the Pannonian Basin.

During the lower Pannonian the fluvial system Palaeo-Danube and its delta were established and entered Lake Pannon in the north-western part of the Vienna Basin. In the delta area huge wetland environments developed, as evidenced by mollusc- and vertebrate-bearing fossil sites such as Gaweinstal and Mariathal (Daxner-Höck 2004b; Harzhauser and Tempfer 2004; Harzhauser et al. 2004; Daxner-Höck et al. 2016).

During the middle Pannonian the wetland areas were destroyed by a last water level rise of Lake Pannon, and at the western margin of the central Vienna Basin pelitic offshore clays were formed during high energy events in Lake Pannon. There, rare fossiliferous layers yielded terrestrial mammals, e. g. in Vösendorf.

In the upper Pannonian, Lake Pannon disappeared successively from the Vienna Basin, and established its north-western coast in the Hungarian Basin (Magyar et al. 1999). Consequently, the drainage systems from the Alps and the Northern Alpine Foreland Basin entered the central Vienna Basin and formed extended floodplains with oxbows, rivulets and floodplain-lakes as reconstructed for the localities Götzendorf and Neusiedl am See (Harzhauser and Tempfer 2004). Simultaneously, a fringe of freshwater lakes became established along the western margin of the central Vienna Basin as suggested for the localities Richardhof-Golfplatz, Richardhof-Wald and Eichkogel (Harzhauser and Tempfer 2004; Daxner-Höck and Höck 2009).

In the western part of Austria, the drainage system of the Alps transported gravels and sands northwards into the Northern Alpine Foreland Basin of Upper Austria. The highest and sratigraphically youngest member of this sequence is the “Hausruck Gravel” (upper Pannonian). Its interbedded sandy-silty layers yielded the Late Miocene vertebrate fauna Schernham (Daxner-Höck 2004a).

The locality Kohfidisch is situated in the Austrian part of the Pannonian Basin (Fig. 1), and represents a karstic cave and fissure system in Devonian limestones, filled with fossiliferous clay of the upper Pannonian /Late Miocene. The locality is well known as the richest fossil deposit of the Pannonian in Austria (Bachmayer and Zapfe 1969; Daxner-Höck and Höck 2015).

## Stratigraphy of Vallesian –Turolian mammal sequences of Austria

The Late Miocene small mammal record of Austria is one of the best in Central Europe. Several key-faunas, important for the understanding of mammal evolution and international correlation are from the Vienna and Pannonian basins. The known localities provide assemblages of stratigraphically relevant mammal/rodent species, and cover an interval from the lower Vallesian (lower Pannonian; Gaweinstal ~11.2 Ma) to the lower Turolian (upper Pannonian; Eichkogel ~ 8.3 Ma). The relative age of the studied localities/faunas and their stratigraphic sequence are well known: Mataschen (Ma), Gaweinstal (Ga), Mariathal (Mat), Vösendorf (Vö), Richardhof-Golfplatz (RH), Götzendorf (Gö), Richardhof-Wald (Rh), Neusiedl am See (NS), Schernham (Sch), Kohfidisch (Ko), Eichkogel (E). (Steininger 1999; Daxner-Höck and Höck 2015; Daxner-Höck et al. 2016).

Most localities also bear mollusc faunas, which can be correlated with the Lake Pannon mollusc biozones A–H, established by Papp (1951) (see also Magyar et al. 1999; Harzhauser et al. 2004).

**Fig. 10:**
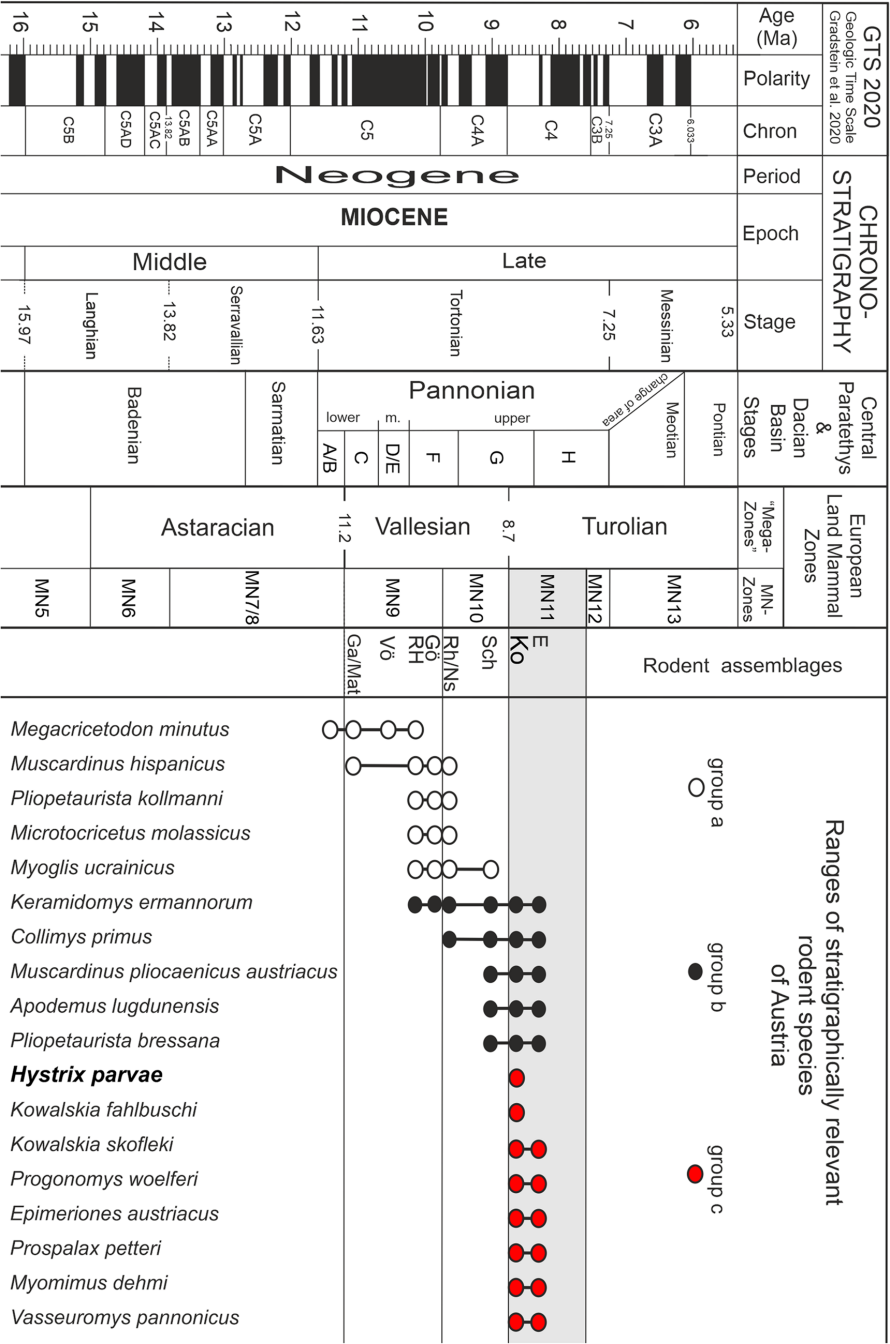
Correlation chart including the Geologic Time Scale (Gradstein et al. 2020), the Miocene Central Paratethys stratigraphy (Piller et al. 2007) and stratigraphy of the Pannonian in the Vienna Basin (Harzhauser et al. 2004), the mollusc letter zones A–H (Papp 1951), the European Land Mammal Zones MN9–MN11 (Steininger 1999), rodent assemblages, and ranges of stratigraphically relevant rodent species of Austria (Daxner-Höck 2004b; Daxner-Höck and Höck 2009, 2015; Daxner-Höck et al. 2016). Selected rodent assemblages are: Eichkogel (*E*), Gaweinstal (*Ga*), Götzendorf (*Gö*), Kohfidisch (*Ko*), Mariathal (*Mat*), Neusiedl am See (*NS*), Richardhof-Golfplatz (*RH*), Richardhof-Wald (*Rh*), Schernham (*Sch*), and Vösendorf (*Vö*)

The selected rodent taxa in group a, b and c (Fig. 10) are characteristic of the Late Miocene in Austria. They provide information about the evolutionary stage, the stratigraphic ranges (first and last occurrences), and serve as index fossils for biostratigraphy and biochronology of Central and Western Europe.

Rodents of group a (five species) range throughout the Vallesian (MN9–MN10), one (*M. minutus*) has its first occurrence as early as upper Astaracian (MN7/8). Rodents of group b range from the Vallesian to the Turolian (upper MN9–MN11 and MN10–MN11). Rodents of group c are limited to the lower Turolian (MN11). Although the faunas of Kohfidisch (Ko) and Eichkogel (E) are composed of groups b and c, the early Turolian age is indicated by species of group **c** with first occurrences in biozone MN11**.** These rodents are: *Hystrix parvae, Kowalskia fahlbuschi, Kowalskia skofleki, Progonomys woelferi, Epimeriones austriacus, Prospalax petteri, Myomimus dehmi* and *Vasseuromys pannonicus* (Fig. 10).

Initially, Kohfidisch (Austria; Bachmayer and Wilson 1970) and Eichkogel (Austria; Daxner-Höck 1980) were recognised as Turolian (MN11) faunas, and correlated with other MN11 faunas of Europe by Weerd van de (1976), i. e. Dorn-Dürkheim (Germany; Franzen and Storch 1975), Crevillente 1-2, Alfambra and Tortajada A (Spain; Weerd 1976), Mollon (France; Michaux 1971). Later, the age of Kohfidisch was thought to be close to the Vallesian/Turolian transition (at ~ 8.7 Ma), and temporarily was changed to the Vallesian (MN10) (De Bruijn et al. 1992: Table 4; Daxner-Höck 1996). Finally, the early Turolian age (MN11) was confirmed by comprehensive studies of the entire small mammal assemblages of Kohfidisch and Eichkogel and other localities of the Late Miocene of Austria (Daxner-Höck 2004a; Ziegler 2006; Daxner-Höck and Höck 2009, 2015; Daxner-Höck et al. 2016). Within the biozone MN11, the estimated age of the Kohfidisch fauna is ~ 8.6 Ma, the younger Eichkogel fauna suggests an age of ~ 8.3 Ma (Daxner-Höck and Höck 2009, Fig. 2; Daxner-Höck and Höck 2015, Fig. 2).

One more fossil site of the Pannonian Basin System, Triblavina, located in the Danube Basin of Slovakia, was identified as lower Turolian (MN11) because it shares *Apodemus lugdunensis, Epimeriones austriacus, Myomimus dehmi, Vasseuromys pannonicus* and other species with Kohfidisch and Eichkogel (Joniak et al. 2020).

The poor fossil record of Csákvár (type locality of *Hystrix parvae*) makes age determination difficult, though the Vallesian/Turolian transition is likely. Weers and Montoya (1996) decided for Vallesian (MN10), and several investigators of *Hystrix* followed this opinion. However, in agreement with Mein (1999) and Kordos (2008) we tend to prefer a Turolian age, because Csákvár shares *H. parvae* with Kohfidisch and Crevillente 2, two faunas with reliable age control (lower Turolian, MN11).

## Conclusions

Detailed investigations of the important fossil material of *Hystrix* parvae from the Austrian locality Kohfidisch shed new light on the morphology of skull and dentition, and allows comparisons with other extinct *Hystrix* species of Europe and Eurasia.

- Here we describe for the first time a fragmentary juvenile skull, five maxilla fragments and thirteen mandible fragments with partial tooth-rows and numerous isolated teeth of different ontogenetic stages, totally more than 100 specimens. The illustrations show photos, SEM-images, microCT-scans of the fragmentary skull and jaws, and isolated cheek-teeth. Moreover, enamel microstructures and cross sections of incisors are shown.
- The cheek teeth are smallest of all extinct species of *Hystrix* s.str., however, occlusal pattern and height show wide ranges, depending on the ontogenetic stage, tooth position and wear stage. Tooth measurements (length, width) of P4/p4 are largest, those of M3/m3 smallest, and the upper P4 and M1/2 are highest. These data of *H. parvae* from Kohfidisch agree with the specimens from Csákvár, Crevillente 2 and Salmendingen.
- The hypsodonty index is calculated as the relationship of enamel height and occlusal length. The upper cheek teeth P4, M1-3 in a low to medium stage of wear (classes A-C) show mean values ranging from 96% to 118%, and therefore are indicated as brachydont to mesodont. D4 and all lower cheek teeth are brachydont, showing mean index values ranging from 33% to 75%.
- Among European species *H. parvae* is smaller than *H. primigenia* (MN11–MN13) and *H. depereti* (MN12– MN15). The mean hypsodonty index of M1/2 (118%) of *H. parvae* partly overlaps with *H. primigenia* (100 to 150%) and is considerably smaller than the younger *H. depereti* (> 150%). Compared with the Asian extinct species, which appear to be stratigraphically younger, the teeth of *H. parvae* are smaller and have lower tooth crowns. By our new data we confirm that *H. parvae* is the smallest and likely oldest of all species of *Hystrix* s.str.
- The fossil record of ten left D4 and ten left p4 evidences a minimum of ten juvenile and ten adult individuals of *H. parvae.* This high percentage of juvenile individuals (~ 50%) suggests favourable nursery environments for *Hystrix* in the cave and fissure system of Kohfidisch.
- The Kohfidisch fauna from the Pannonian of the Central Paratethys correlates with the lower Turolian as confirmed by several first occurrences of rodents, *Hystrix parvae, Kowalskia fahlbuschi, Kowalskia skofleki, Progonomys woelferi, Epimeriones austriacus, Prospalax petteri, Myomomus dehmi* and *Vasseuromys pannonicus.* The fauna is correlative with the Late Miocene (MN11), and suggests an age of ~ 8.6 Ma.

## Supplementary Information

Below is the link to the electronic supplementary material.

Supplementary file1 (DOCX 23 KB)

## Data Availability

All data generated or analysed during this study are included in this published article. All fossils and the according collection numbers are listed in the material list and in the Appendix.
