# Supplementary material for: The porcupine Hystrix parvae (Kretzoi, 1951) from the Late Miocene (Turolian, MN11) of Kohfidisch in Austria
Source: Paleobiodivers Paleoenviron. 2024 Aug 3;105(1):313–34. doi: 10.1007/s12549-024-00616-3 (PMC12011660; doi:10.1007/s12549-024-00616-3)
Supplement: Supplementary file 1 — Supplementary file1 (DOCX 23 KB) [file 12549_2024_616_MOESM1_ESM.docx]

**Appendix**

| NHMW 2011/0113/0001 | skull frag, juv | left | I2, D4-M1-M2 | Ko-IIIo |
| --- | --- | --- | --- | --- |
| NHMW 2011/0113/0001 | skull frag, juv | right | I2, D4- M1-M2 | Ko-IIIo |
| NHMW 2011/0113/0002 | maxilla frag, juv | left | M2-M3 | Ko-IIIo |
| NHMW 2011/0113/0003 | maxilla frag, juv | left | D4-M1 | Ko-IIIu |
| NHMW 2011/0113/0004 | maxilla frag, juv | left | D4-M1 | Ko-IIIu |
| NHMW 2011/0113/0005 | maxilla frag, juv | left | D4-M1-M2 | Ko |
| NHMW 2011/0113/0006 | isolated tooth | right | M1 | Ko-III |
| NHMW 2011/0113/0007 | isolated tooth | right | D4 | Ko |
| NHMW 2011/0113/0008 | isolated tooth | right | D4 | Ko-IIIo |
| NHMW 2011/0113/0009 | isolated tooth | left | D4 | Ko-IIIu |
| NHMW 2011/0113/0010 | isolated tooth | left | D4 | Ko |
| NHMW 2011/0113/0011 | isolated tooth | left | D4 | Ko |
| NHMW 2011/0113/0012 | isolated tooth | left | D4 | Ko-IIIu |
| NHMW 2011/0113/0013 | isolated tooth | left | D4 | Ko-IIIo |
| NHMW 2011/0113/0014 | isolated tooth | left | P4 | Ko-IIIu |
| NHMW 2011/0113/0015 | isolated tooth | left | P4 | Ko-IIIu |
| NHMW 2011/0113/0016 | isolated tooth | left | p4 | Ko-IIIu |
| NHMW 2011/0113/0017 | isolated tooth | right | P4 | Ko-IIIo |
| NHMW 2011/0113/0018 | isolated tooth | right | P4 | Ko-I |
| NHMW 2011/0113/0019 | isolated tooth | right | M1/2 | Ko-IIIu |
| NHMW 2011/0113/0020 | isolated tooth | right | M1/2 | Ko-I |
| NHMW 2011/0113/0021 | isolated tooth | right | M1/2 | Ko-I |
| NHMW 2011/0113/0022 | isolated tooth | left | M1/2 | Ko-IIIo |
| NHMW 2011/0113/0023 | isolated tooth | left | M1/2 | Ko-IIIo |
| NHMW 2011/0113/0024 | isolated tooth | left | M1/2 | Ko-IIIo |
| NHMW 2011/0113/0025 | isolated tooth | left | M2 | Ko-Cm |
| NHMW 2011/0113/0026 | isolated tooth | left | M1/2 | Ko-IIIo |
| NHMW 2011/0113/0027 | isolated tooth | left | M1/2 | Ko-IIIo |
| NHMW 2011/0113/0028 | isolated tooth | left | M1/2 | Ko-IIIu |
| NHMW 2011/0113/0029 | isolated tooth | left | M1/2 | Ko-IIIu |
| NHMW 2011/0113/0030 | isolated tooth | left | M1/2 | Ko-IIIu |
| NHMW 2011/0113/0031 | isolated tooth | right | M1/2 | Ko-IIIu |
| NHMW 2011/0113/0032 | isolated tooth | right | M1/2 | Ko |
| NHMW 2011/0113/0033 | isolated tooth | right | M1/2 | Ko |
| NHMW 2011/0113/0034 | isolated tooth | right | M1/2 | Ko |
| NHMW 2011/0113/0035 | isolated tooth | right | M1/2 | Ko-IIIu |
| NHMW 2011/0113/0036 | isolated tooth | right | M1/2 | Ko |
| NHMW 2011/0113/0037 | isolated tooth | right | M1/2 | Ko |
| NHMW 2011/0113/0038 | isolated tooth | right | M3 | Ko-IIIu |
| NHMW 2011/0113/0039 | isolated tooth | right | M3 | Ko-I |
| NHMW 2011/0113/0040 | isolated tooth | left | M3 | Ko-IIIo |
| NHMW 2011/0113/0041 | isolated tooth | left | M3 | Ko-I |
| NHMW 2011/0113/0042 | isolated tooth | right | M3 | Ko-IIIu |
| NHMW 2011/0113/0043 | isolated tooth | right | M3 | Ko-IIIu |
| NHMW 2011/0113/0044 | isolated tooth | right | M3 | Ko |
| NHMW 2011/0113/0045 | isolated tooth | right | M3 | Ko |
| NHMW 2011/0113/0046 | isolated tooth | right | M3 | Ko |
| NHMW 2011/0113/0047 | isolated tooth | right | M3 | Ko |
| NHMW 2011/0113/0048 | mandible, frag | left | p4-m1-m2-m3 | Ko-III |
| NHMW 2011/0113/0049 | mandible, frag, juv | right | d4-m1 | Ko-III |
| NHMW 2011/0113/0050 | mandible, frag | right | m1-m2-m3 | Ko-IIIu |
| NHMW 2011/0113/0051 | mandible, frag | right | m1-m2-m3 | Ko-IIIu |
| NHMW 2011/0113/0052 | mandible, frag, juv | left | d4-m1-m2-m3 | Ko-IIIu |
| NHMW 2011/0113/0053 | mandible, frag, juv | left | d4-m1-m2 | Ko-IIIu |
| NHMW 2011/0113/0054 | mandible, frag, juv | left | d4-m1-m2 | Ko-IIIu |
| NHMW 2011/0113/0055 | mandible, frag | right | m1-m2-m3 | Ko-I |
| NHMW 2011/0113/0056 | mandible, frag | left | p4, m2 | Ko-I |
| NHMW 2011/0113/0057 | mandible, frag | right |  | Ko-I |
| NHMW 2011/0113/0058 | isolated tooth | left | d4 | Ko-IIIu |
| NHMW 2011/0113/0059 | isolated tooth | left | d4 | Ko-IIIu |
| NHMW 2011/0113/0060 | isolated tooth | left | d4 | Ko-IIIu |
| NHMW 2011/0113/0061 | isolated tooth | left | d4 | Ko-IIIu |
| NHMW 2011/0113/0062 | isolated tooth | right | d4 | Ko-IIIu |
| NHMW 2011/0113/0063 | isolated tooth | right | d4 | Ko-IIIu |
| NHMW 2011/0113/0064 | isolated tooth | left | p4 | Ko-IIIu |
| NHMW 2011/0113/0065 | isolated tooth | left | p4 | Ko-III |
| NHMW 2011/0113/0066 | isolated tooth | left | p4 | Ko-IIIo |
| NHMW 2011/0113/0067 | isolated tooth | left | p4 | Ko-IIIo |
| NHMW 2011/0113/0068 | isolated tooth | left | p4 | Ko-IIIo |
| NHMW 2011/0113/0069 | isolated tooth | left | p4 | Ko-IIIu |
| NHMW 2011/0113/0070 | isolated tooth | left | p4 | Ko-I |
| NHMW 2011/0113/0071 | isolated tooth | left | p4 | Ko-I |
| NHMW 2011/0113/0072 | isolated tooth | left | p4 | Ko-IIIu |
| NHMW 2011/0113/0073 | isolated tooth | right | p4 | Ko-IIIu |
| NHMW 2011/0113/0074 | mandible frag | left | m1 | Ko-IIIo |
| NHMW 2011/0113/0075 | isolated tooth | left | m1 | Ko-IIIu |
| NHMW 2011/0113/0076 | isolated tooth | left | m1/2 | Ko-Cm |
| NHMW 2011/0113/0077 | isolated tooth | left | m1/2 | Ko-III |
| NHMW 2011/0113/0078 | isolated tooth | left | m1/2 | Ko-IIIu |
| NHMW 2011/0113/0079 | isolated tooth | left | m1/2 | Ko-IIIo |
| NHMW 2011/0113/0080 | isolated tooth | right | m1 | Ko-IIIo |
| NHMW 2011/0113/0081 | isolated tooth | right | m1/2 | Ko-IIIo |
| NHMW 2011/0113/0082 | isolated tooth | right | m1/2 | Ko-I |
| NHMW 2011/0113/0083 | isolated tooth | right | m1/2 | Ko-I |
| NHMW 2011/0113/0084 | isolated tooth | left | m1/2 | Ko-IIIu |
| NHMW 2011/0113/0085 | mandible frag | left | m2 | Ko-Cm |
| NHMW 2011/0113/0086 | isolated tooth | left | m1/2 | Ko-IIIu |
| NHMW 2011/0113/0087 | isolated tooth | left | m3 | Ko-IIIu |
| NHMW 2011/0113/0088 | isolated tooth | left | m3 | Ko-IIIu |
| NHMW 2011/0113/0089 | isolated tooth | left | m3 | Ko-IIIo |
| NHMW 2011/0113/0090 | isolated tooth | left | m3 | Ko-IIIo |
| NHMW 2011/0113/0091 | isolated tooth | right | m3 | Ko-IIIu |
| NHMW 2011/0113/0092 | isolated tooth | right | m3 | Ko-I |
| NHMW 2011/0113/0093 | isolated tooth | right | m3 | Ko-IIIo |
| NHMW 2011/0113/0094 | isolated tooth | left | M | Ko-IIIu |
| NHMW 2011/0113/0095 | isolated tooth | right | M | Ko-IIIu |
| NHMW 2011/0113/0096 | isolated tooth | left | M2 | Ko-IIIu |
| NHMW 2011/0113/0097 | isolated tooth | left | D4 | Ko-IIIo |
| NHMW 2011/0113/0098 | maxilla frag | right | P4-M1-M2 | Ko-IIIb  1983-IIIb |
| NHMW 2011/0113/0099 | mandible frag | left | i2-m1-m2-m3 | Ko-I  1982-1 |
| NHMW 2011/0113/0100 | isolated teeth |  | 3 incisors frag | Ko-I |
| NHMW 2011/0113/0100 | isolated teeth |  | 4 incisors frag | Ko-II |
| NHMW 2011/0113/0100 | isolated teeh |  | 5 incisors frag | Ko-III |
| NHMW 2011/0113/0100 | isolated teeh |  | 20 incisors frag | Ko-IIIo |
| NHMW 2011/0113/0100 | isolated teeth |  | 30 incisors frag | Ko-IIIu |
| NHMW 2011/0113/0100 | isolated teeth |  | 7 incisors frag | Ko-Cm |
| NHMW 2011/0113/0101 | isolated tooth |  | 1 incisor sup frag | Ko-I |
| NHMW 2011/0113/0102 | isolated tooth |  | 1 incisor inf frag | Ko-I |
| NHMW 2011/0113/0103 | isolated tooth |  | 1 incisor frag | Ko-II |
| NHMW 2011/0113/0104 | isolated tooth |  | 1 incisor frag | Ko-II |

*Hystrix parvae* (Kretzoi, 1951) from Kohfidisch, Austria, Late Miocene, Turolian (MN11).

The specimen list contains: collection numbers (NHMW 2011/0113/0001-0104) of fragmentary skull, maxilla fragments, mandible fragments and isolated teeth, the body side (left, right), tooth position (D4, M1….), and localization (Ko-I, Ko-II, Ko-III, Ko-IIIo, Ko-IIIu, Ko-Cm).
